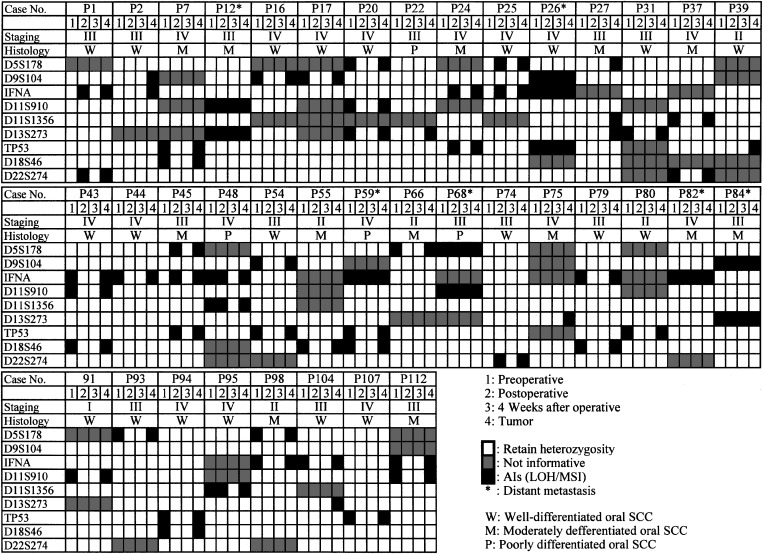# Monitoring of circulating tumour-associated DNA as a prognostic tool for oral squamous cell carcinoma

**DOI:** 10.1038/sj.bjc.6602635

**Published:** 2005-05-31

**Authors:** K Hamana, K Uzawa, K Ogawara, M Shiiba, H Bukawa, H Yokoe, H Tanzawa

**Affiliations:** 1 Department of Clinical Molecular Biology, Graduate School of Medicine, Chiba University, 1-8-1 Inohana, Chuo-ku, Chiba 260-8670, Japan; 2 Division of Dentistry and Oral-Maxillofacial Surgery, Chiba University Hospital, 1-8-1 Inohana, Chuo-ku, Chiba 260-8670, Japan; 3 Center of Excellence (COE) Program in the 21st Century, Graduate School of Medicine, Chiba University, 1-8-1 Inohana, Chuo-ku, Chiba 260-8670, Japan

**Keywords:** oral squamous cell carcinoma, allelic imbalance, serum DNA, circulating tumour DNA, prognosis

## Abstract

Frequent allelic imbalances (AIs) including loss of heterozygosity and microsatellite instability on a specific chromosomal region have been identified in a variety of human malignancies. The objective of our study was to assess the possibility of prognostication and monitoring of oral squamous cell carcinoma (SCC) by microsatellite blood assay. DNA from normal and tumorous tissues and serum DNA obtained at three time points (preoperatively, postoperatively, and 4 weeks postoperatively) from 64 patients with oral SCC was examined at nine microsatellite loci. In all, 38 (59%) DNA samples from tumorous tissues and 52% from serum showed AIs in at least one locus. Patterns of AIs in the serum DNA were matched to those detected in tumour DNA. Of them, AIs were frequently detected preoperatively (44%, 28 of 64), and postoperatively (20%, 13 of 64). Moreover, among 12 cases with AIs during the postoperative period, six had no evidence of an AI 4 weeks postoperatively, and they had no recurrence and were disease free. In contrast, six patients with AI-positive DNA 4 weeks postoperatively have died with distant metastasis within 44 weeks. Thus, our results suggest that the assessment of microsatellite status in the serum DNA could be a useful predictive tool to monitor disease prognosis.

Squamous cell carcinoma (SCC), which contributes to more than 90% of all malignant tumours of the oral cavity, is characterised by regional and distant metastases. Most patients with oral SCC who have either suspected or proven metastases in the regional lymph nodes are candidates for composite resection in which the lesion, surrounding tissues, and lymph nodes of the neck are all removed. Since oral tumours, if undetected, frequently may metastasise to solitary lung tumours, early and sensitive detection of the metastasis is of primary importance and requires close surveillance for successful treatment. Despite its clinical importance, at present there is no predictive procedure related to the recurrence, metastasis, or both.

It is widely accepted that the accumulation of genetic damage is a major cause of the development of human malignancies including oral SCC (Scully *et al*, 2000; Williams, 2000). Thus, DNA can provide one of the most direct sources for potential markers. As sample materials for diagnosis should be easily accessible by a minimally invasive procedure, there has been much interest in the potential use of nucleic acid markers in the blood of patients with cancer. Allelic imbalances (AIs) appearing as loss of heterozygosity (LOH) or as microsatellite instability (MSI) have been detected in the circulating DNA of patients with a variety of malignancies, such as non-small-cell lung cancer (Sozzi *et al*, 1999), renal cell carcinoma (Gonzalgo *et al*, 2002), bladder cancer (Utting *et al*, 2002), breast cancer (Silva *et al*, 1999), colon cancer (Diep *et al*, 2003), and malignant melanoma (Taback *et al*, 2004). Nawroz *et al* (1996) first demonstrated that AIs could be detected in the plasma/serum DNA of head and neck SCC, suggesting that circulating tumour-associated DNA in the blood of patients with oral SCC can be a key determinant in predicting tumour recurrences or metastasis.

In the present study, we evaluated the microsatellite status of serum-isolated DNA preoperatively, postoperatively, and 4 weeks postoperatively from patients with oral SCC using nine microsatellite markers recently reported to be frequent loci showing AIs in primary oral SCCs.

## MATERIALS AND METHODS

### Patients

In all, 64 patients (38 males and 26 females; mean age, 68 years; range, 39–81 years) who underwent primary oral SCC resection at the Division of Dentistry and Oral-Maxillofacial Surgery, Chiba University Hospital from 2000 to 2004 were selected to participate in the present study. No patients underwent a blood transfusion. Informed consent was obtained from all patients and the patients' families, and our protocol was reviewed and approved by the institutional review board of Chiba University.

Histopathologic diagnosis of each neoplastic tissue was performed according to the World Health Organization criteria by the Department of Pathology, Chiba University Hospital, and all resected tumours had been histopathologically diagnosed with tumour-free margins. Clinicopathologic staging was determined by the TNM classification of the International Union against Cancer.

Among the 64 patients with oral SCC, three were in stage I, 14 in stage II, 23 in stage III, and 25 in stage IV. Prospective follow-up, starting after surgery, and diagnosis was based on regular (every month during the first year and every 3 months during the second year) clinical, biochemical, and radiologic examinations, including bone scans.

### DNA isolation

Peripheral blood (5 ml) samples were collected preoperatively, postoperatively, and 4 weeks postoperatively. Blood samples were centrifuged in EDTA tubes for 10 min at 3000 **g** to obtain the serum from the supernatant. Tumour tissues were obtained at the time of surgical resection. Resected tissues were immediately snap-frozen in liquid nitrogen. The peripheral lymphocytes of each patient were used as a source of normal DNA.

DNA was extracted from tissue, sera, and lymphocytes using a QIAamp Blood and Tissue Kit (Qiagen, Hilden, Germany) as recommended by the manufacturer. The extracted DNA was eluted in 100 *μ*l of sterile water and stored at −20°C until analysis.

### Microsatellite analysis

Nine microsatellite markers on seven chromosome arms were selected for high polymorphism, small size of amplified fragment, and location at sites frequently undergoing AIs in oral SCC. The following microsatellite markers purchased from Research Genetics (Huntsville, AL, USA) were selected based on previous reports (Uzawa *et al*, 1994, 1996; Gonzalez *et al*, 1995; Watanabe *et al*, 1997; Miyakawa *et al*, 1998; Ogawara *et al*, 1998; Nakanishi *et al*, 1999): D5S178 (5q21), D9S104 (9p21), IFNA (9p22), D11S910 (11q23), D11S1356 (11q25), D13S273 (13q14–21), TP53 (17p13), D18S46 (18q21), and D22S274 (22q13). Polymerase chain reaction (PCR) amplification was carried out in a 25-*μ*l final volume containing 0.6 U of *Taq* polymerase in PCR buffer (50 mM KCl, 10 mM Tris-Cl (pH 8.0), 1.5 mM MgCl_2_), 80 *μ*M of each dNTP, and 10 pmol of each primer. Samples were processed through 32 cycles, with each cycle consisting of 1 min at 94°C, 1 min at an annealing temperature of 55–57°C (as appropriate for each primer), and 1 min at 72°C. A negative control without DNA was included in every PCR series. The amplified PCR products were loaded in parallel on 5% polyacrylamide gel containing 7 M urea and visualised by silver staining.

The normal pattern at each microsatellite in each individual was defined as the pattern in each corresponding normal tissue. Loss of heterozygosity was assessed by scanning densitometry, analysed by National Institutes of Health (NIH) software (Image version 1.62, Dr W Rasband, NIH, Bethesda, MD, USA), and then diagnosed as the signal intensity in cancer-associated DNA, that is, less than 30% of that of control DNA from the identical patient. Alterations were designated as MSI when any band that was not seen in normal DNA appeared in tumour-associated DNA. Duplicate examinations were performed to confirm AIs in each subject.

### Statistical analysis

Significant differences were calculated by Fisher's exact test. One-tailed *P*-values less than 0.05 were considered statistically significant.

## RESULTS

In all, 192 blood samples were collected from 64 subjects at the three time points. In addition to sera, genomic DNA from 64 pairs of tumour samples and normal controls were isolated. Overall, 320 DNA samples were evaluated by microsatellite analysis.

Using a panel of nine microsatellite markers, we checked for the presence of tumour-associated DNA in the serum by comparing the markers with each normal control in the 64 patients with oral SCC. Sporadic AIs in DNA from tumour tissues were observed in 59% (38 of 64) of the patients in at least one or more loci examined. Table 1 shows microsatellite alteration status in all patients with AIs (*n*=38). Of them, five patients had only one AI in tumour tissue DNA without AI in the serum obtained at any time point. Therefore, it was possible to detect at least one AI in the serum DNA in 87% (33 of 38) of the patients with AI in tumour DNA, suggesting the presence of circulating tumour DNA. All AI patterns in sera were matched to those detected in tumour DNA from the identical patients, indicating that the presence of AI in the serum was associated with the presence of AI in paired tumour. Free-circulating tumour-associated DNA was detected preoperatively (44%, 28 of 64) and postoperatively (20%, 13 of 64). Among the patients with AI preoperatively (*n*=28), 20 (71%) had no evidence of AI in sera postoperatively, they have had no recurrence or distant metastasis, and are disease free. In contrast to AI-free patients, two patients with AI-positive DNA 4 weeks postoperatively died with distant metastasis within 44 weeks after surgery.

We detected a significant correlation between the presence of AIs in the serum DNA and tumour stage (*P*=0.0378, stage I/II *vs* stage III/V). However, the presence of serum DNA alterations did not correlate with other clinical parameters, such as tobacco use, alcohol consumption, age, tumour differentiation, or lymph node status.

**Figure 1:**
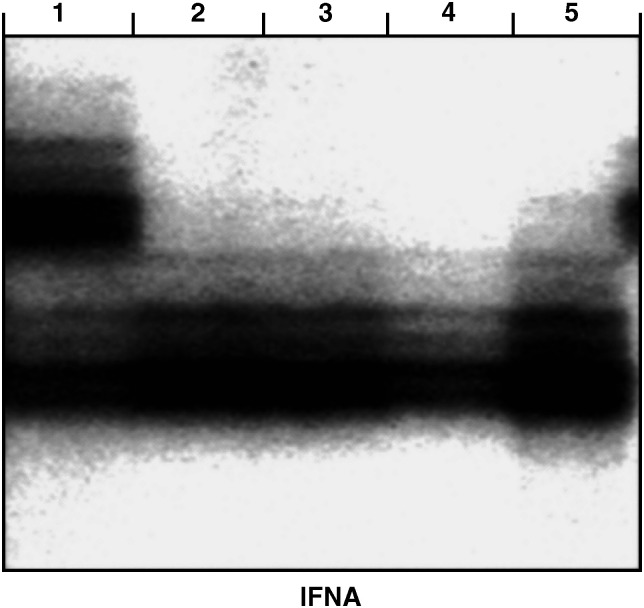
Representative results of the microsatellite analysis at the IFNA locus of a lymphocyte (lane 1), preoperative sera (lane 2), postoperative sera (lane 3), sera 4 weeks postoperatively (lane 4), and tumour (lane 5) from a patient with oral SCC (case P24). While DNA from lymphocyte reveals two major silver-stained bands, LOH of top alleles are detected in DNA from sera at all time points and from tumour tissue.

The most frequently altered chromosome in the serum DNA was at the IFNA locus on 9p21 (40%, 15 of 38), where *p16* and *p15* tumour suppressor genes are in close proximity. All IFNA AIs were observed in patients with oral SCC classified as stage III or IV disease with the exception of one stage II case. Typical examples of the microsatellite analysis are shown in Figure 1.

## DISCUSSION

The circulating DNA in the plasma or serum of patients with cancer has been shown to reflect the characteristics of the tumour DNA including molecular changes, such as methylation, point mutations, and MSI. The first description regarding the use of microsatellite polymorphic markers to identify and characterise circulating tumour DNA in patients with head and neck SCCs, including oral SCCs, was that of Nawroz *et al* (1996). A number of recent reports suggested that tumour-associated DNA in the serum may be a tool for early diagnosis or prognosis in a wide variety of cancers. In the current study, we evaluated the hypothesis that detecting circulating tumour-associated DNA using tumour-specific AIs might have clinical use in oral SCC, and our data on oral SCC suggest that the detection of free-circulating tumour-associated DNA during the postoperative period may be of prognostic value.

The sensitivity of microsatellite analysis in the serum from head and neck SCC including oral SCC has differed from study to study. For example, while Coulet *et al* (2000) found that only 2% of patients with head and neck cancer had AIs, higher incidences of AIs were detected (between 18.7 and 45%) (Nunes *et al*, 2001; Nawroz-Danish *et al*, 2004).

We carried out microsatellite analysis using nine markers on serum samples obtained at three time points in 64 patients with oral SCC. In all 52% (33 of 64) of patients had AIs for at least one or more loci or period in their serum identical to tumour DNA. Since we selected serum samples at different time points with high polymorphic markers, the high prevalence of AIs could be detected in this study.

We also found that there was a strong statistical correlation between the presence of AIs in the serum DNA and tumour stage (*P*<0.05, stage I/II *vs* stage III/V), which has been considered one of the most important prognostic determinants. The detection of AIs in the serum should be considered an attractive approach for predicting the prognosis of patients with oral SCC.

The most frequent marker with AI was the IFNA locus at 9p21, where *p16* and *p15* tumour suppressor genes frequently mutated, deleted, and methylated in various types of human cancers are in close proximity. In addition, vast majority of AIs at the IFNA locus were observed in advanced oral SCCs (stage III or IV), suggesting that this chromosomal abnormality possibly associated with the inactivation of *p16* and *p15* may contribute to the progression of this disease. In this context, the presence of a chromosomal deletion at the 9p21 locus has been reported to be linked to local, regional, or distant recurrence in head and neck SCCs (Lydiatt *et al*, 1998).

It is of interest to note that while most AI-positive patients (84%, 32 of 38) had lost the tumour-associated DNA in their serum by 4 weeks postoperatively, AI in the serum DNA still was detected in six patients 4 weeks after surgery, and those patients had a poor prognosis with distant metastasis. Furthermore, Figure 1 also shows that the AI was detected not only in tumour DNA but also in the serum at all time points. At present, we have no exact explanation as to why these patients still had AIs even at the last time point examined. However, the proposed mechanisms may include: (1) differences in activation of their tumour cells; (2) differences in the number of tumour cells; and (3) decline of their autoimmune systems.

In conclusion, based on this pilot study of 64 patients with nine markers, microsatellite analysis at three time points in their treatment may predict the risk of future recurrence and death. Thus, we propose that the microsatellite blood assay should be considered as a clinically important monitoring tool for assessing patient response to adjuvant therapy and in the surveillance of patients who are clinically disease free for the earliest signs of recurrence or distant metastasis. Larger prospective randomised trials are needed to validate the clinical utility of our findings.

## Figures and Tables

**Table 1:** Microsatellite status of patients with OSCCs